# Magnetosheath Jet Formation Influenced by Parameters in Solar Wind Structures

**DOI:** 10.1029/2023JA031339

**Published:** 2023-04-07

**Authors:** Florian Koller, Ferdinand Plaschke, Manuela Temmer, Luis Preisser, Owen W. Roberts, Zoltan Vörös

**Affiliations:** ^1^ Institute of Physics University of Graz Graz Austria; ^2^ Institut für Geophysik und Extraterrestrische Physik TU Braunschweig Braunschweig Germany; ^3^ Space Research Institute Austrian Academy of Sciences Graz Austria; ^4^ Institute of Earth Physics and Space Science ELRN Sopron Hungary

**Keywords:** magnetosheath jets, low jet generation in CMEs, CMEs disrupt foreshock building, high jet generation in high speed streams, high speed streams provide ideal conditions for jets, SW parameter investigation of jet modulation

## Abstract

Magnetosheath jets are dynamic pressure enhancements observed in the terrestrial magnetosheath. Their generation mechanisms are currently debated but the majority of jets can be linked to foreshock processes. Recent results showed that jets are less numerous when coronal mass ejections (CMEs) cross the magnetosheath and more numerous when stream interaction regions (SIRs) cross it. Here, we show for the first time how the pronounced substructures of CMEs and SIRs are related to jet production. We distinguish between compression and magnetic ejecta (ME) regions for the CME as well as compression region associated with the stream interface and high‐speed streams (HSSs) for the SIR. Based on THEMIS and OMNI data covering 2008–2021, we show the 2D probability distribution of jet occurrence using the cone angle and Alfvén Mach number. We compare this distribution with the values within each solar wind (SW) structure. We find that both high cone angles and low Alfvén Mach numbers within CME‐MEs are unfavorable for jet production as they may inhibit a well‐defined foreshock region. 1D histograms of all parameters show, which SW parameters govern jet occurrence in each SW structure. In terms of the considered parameters the most favorable conditions for jet generation are found for HSSs due to their associated low cone angles, low densities, and low magnetic field strengths.

## Key Points

- We show how plasma parameters in solar wind structures are related with jet occurrence
- High cone angles and low Alfvén Mach numbers associated with the magnetic ejecta of coronal mass ejections presumably reduce the generation of jets
- We found that plasma parameters in high‐speed streams represent the most favorable physical condition for jet generation

## 1 Introduction

The magnetosheath is the region of shocked solar wind (SW) plasma sunward of the Earth's magnetosphere. Němeček et al. (1998) first reported that the magnetosheath regularly shows structures with dynamic pressure enhancements, which we shall call jets in the present work. Jets can show an increase in dynamic pressure up to 15 times compared to the surrounding plasma (Plaschke et al., 2013). Their median size is estimated to be 0.1 R_*e*_ but can reach up to more than 2 R_*e*_ (Plaschke et al., 2016, 2020). Large jets in particular can be geoeffective (Hietala et al., 2018; Norenius et al., 2021; Nykyri et al., 2019) and appear several times per hour (Plaschke et al., 2016).

Recently, several generation mechanisms were proposed to explain the occurrence of magnetosheath jets (Plaschke et al., 2018). Most mechanisms explain jets as a result of different processes in the foreshock region and are therefore associated with the quasi‐parallel bow shock. The foreshock can only build up due to back‐streaming ions from a super‐critical bow shock and is therefore dependent on a high Alfvén Mach number (Balogh & Treumann, 2013), which is defined as *M*_*A*_ = *v*_*sw*_/*v*_*A*_, with *v*_*sw*_ denoting the SW velocity, and $v_{A}=B/\sqrt{\mu_{0}\rho }$ (with B being the magnetic field strength, *μ*_0_ the magnetic permeability and *ρ* the SW density, respectively) defining the Alfvén velocity. The foreshock is formed upstream of the quasi‐parallel shock front and therefore requires a low shock normal angle Θ_*Bn*_ (Eastwood et al., 2005). The interplanetary magnetic field (IMF) cone angle (arccos |*B*_*x*_|/|*B*|, with *B*_*x*_ denoting the magnetic field strength in GSE‐X, with GSE being the geocentric solar ecliptic coordinate system) is often used as a good approximation for Θ_*Bn*_ for the subsolar region (see e.g., Plaschke et al., 2013; Raptis et al., 2020; Vuorinen et al., 2019). Θ_*Bn*_ is identical to the cone angle at the subsolar point at the nose of the bow shock. Bow shock models are required to get Θ_*Bn*_ at positions far away of the subsolar point due to the curved geometry of the shock.

It has been shown that jets appear more often during low cone angle periods (Gutynska et al., 2015; LaMoury et al., 2021; Plaschke et al., 2013; Vuorinen et al., 2019). Different phenomena in the foreshock can cause ripples in the bow shock (Balogh & Treumann, 2013). The way in which the SW is processed by the rippled shock has been proposed to be the cause for jet generation (Hietala et al., 2009; Hietala & Plaschke, 2013; Preisser et al., 2020). At the ripple, the local oblique shock front may cause the deceleration of the incoming SW plasma to be less efficient in the GSE‐X direction in comparison to the less oblique shock surroundings. It would create a flow (jet) in the downstream side of the shock that is faster than the surrounding shocked and decelerated plasma. This effect as well as the integration of fast foreshock flows into the magnetosheath might also be a consequence of short large‐amplitude magnetic field structures (SLAMS) forming in the foreshock (Karlsson et al., 2015; Palmroth et al., 2018b; Schwartz & Burgess, 1991). The latest simulations have shown that the majority of jets can be related to foreshock compressional structures (Suni et al., 2021). Recently, Raptis, Karlsson, Vaivads, Pollock, et al. (2022) presented evidence that jets can be generated as a consequence of the bow shock reformation process at the quasi‐parallel shock front itself. This has been also proposed to be a mechanism for the formation of paramagnetic embedded plasmoids based on hybrid simulations (Preisser et al., 2020). Hietala and Plaschke (2013) estimated that the majority (97%) of jets can be associated to bow shock rippling. A subset of jets can be explained by other mechanisms. For example, Archer et al. (2012) suggested that rotational discontinuities in the magnetic field cause pressure pulses in the magnetosheath when there is a change from the quasi‐parallel to the quasi‐perpendicular shock region and vice‐versa.

In a recent statistical study Koller et al. (2022) analyzed jet occurrence within large scale SW structures, such as coronal mass ejections (CMEs) and stream interaction regions (SIRs) together with their high‐speed streams (HSSs). It was found that jets are less frequent when the magnetic ejecta (ME) region of the CME passes Earth.

**Figure 1:**
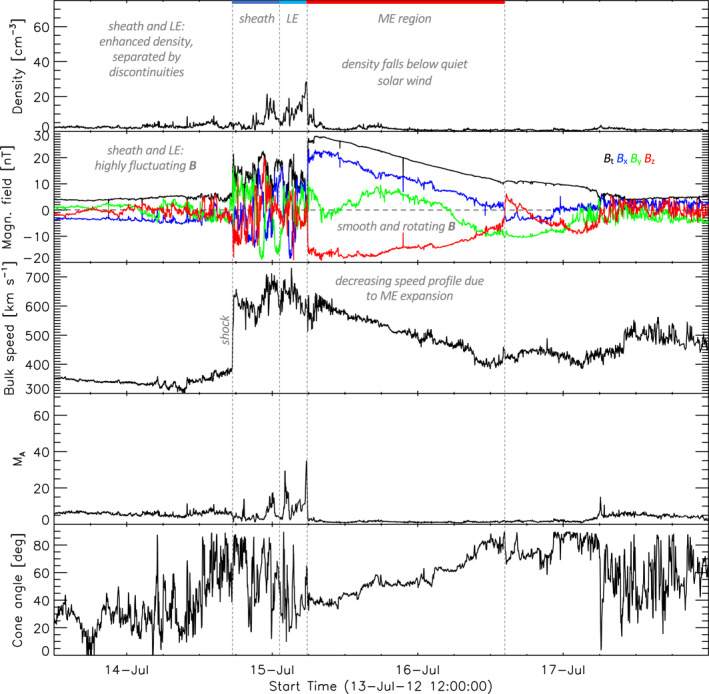
Example of a coronal mass ejection (CME) from active composition explorer measurements. The three panels from top to bottom show the measured proton density, total magnetic field and vector components in geocentric solar ecliptic coordinates (see legend), and the proton bulk speed. This CME clearly reveals the typical structures, shock, two density enhancements—sheath (dark blue) and leading edge (LE, light blue)—separated from each other by discontinuities and forming the highly turbulent compression region. The LE is followed by the magnetic ejecta (ME) with the twisted magnetic field components. The next two panels give the Alfvén Mach number and the cone angle (see more details in the text).

In comparison to quiet SW conditions and compressed SW for SIRs, CMEs and their ME regions present “laboratories” with very different SW conditions. Typically, when CMEs exceed the speed of the fast magnetosonic wave they drive a shock and behind it the CME‐sheath region is built up showing an increase in velocity, density and magnetic field (see e.g., Kilpua et al., 2017). Recent results revealed, that the sheath region covers two separate density structures of compressed and piled up SW, namely the sheath and leading edge (LE) (see Temmer & Bothmer, 2022), which is driven by the strong and smoothly rotating magnetic flux rope. The CME compression region (sheath and LE) is, in contrast to the flux rope, very turbulent and the cone angle can change rapidly within a short time. Figure 1 shows a CME example measured by the Active Composition Explorer (ACE, Stone et al., 1998). In the present work, we therefore investigate on the basis of these recent results the physical mechanism of the decrease in jet occurrence of jets. The results will give us a better understanding of jet production mechanisms. We hypothesize that the conditions inside the CME‐ME pose difficulties for the building of a foreshock in the subsolar region that can produce highly non‐linear structures. Due to the twisted magnetic field lines in the flux rope inside of the ME, the cone angle could differ greatly from radial IMF conditions. Based on cone angle measurements, Turc et al. (2016) showed that during the majority of magnetic clouds that arrived at Earth, the subsolar bow shock configuration is quasi‐perpendicular. Radial IMF lines however seem to be a necessary condition to generate a quasi‐parallel shock region that builds the foreshock. In addition to that, the high magnetic field strength and low density inside a CME‐ME cause an increase in Alfvén velocity. Thus, the Alfvén Mach number decreases, causing a decrease in the strength of the bow shock (see Lavraud & Borovsky, 2008). The sum of all these effects generated by the arrival of the CME‐ME to the bow shock could inhibit the building of a foreshock region that can efficiently generate jets near the subsolar point.

**Figure 2:**
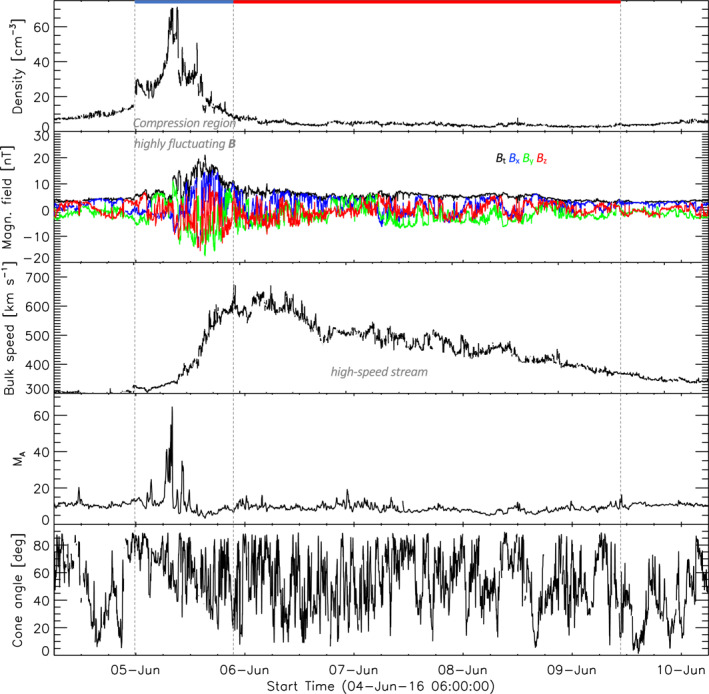
Example of an stream interaction region and high‐speed stream from OMNI measurements. The panels show the same parameters as the previous figure.

Similar to CME‐sheaths, SIRs consist of compressed SW due to HSSs originating from coronal holes (Jian et al., 2006; Temmer, 2021). Figure 2 shows an example of a SIR and its HSS using OMNI data (King & Papitashvili, 2005). The SIR compression region is turbulent with strongly increased density and magnetic field. HSSs that drive the compression show a fast flow speed and low density. Unlike CME‐MEs, the magnetic field strength inside HSSs does not exhibit an exceptionally enhanced structure. Because of this combination of parameters, high Mach numbers are expected within the HSSs. Low cone angles are statistically expected to appear more often compared to quiet SW due to the fast flow speed. SIRs last for several days and are often reoccurring after a Solar rotation (which is then called a co‐rotating interaction region). The conditions for building up a foreshock near the subsolar point should be met often inside HSSs. Koller et al. (2022) found that the occurrence of jets is increased especially after the velocity peak of the HSS.

In the following we investigate jets detected by THEMIS spacecraft between 2008 and 2021 and compare the SW conditions during these times. In this way, we will identify which parameters govern jet production in each SW structure. Initially we consider two parameters that are indicative for the existence of the foreshock near the subsolar point: cone angle and Alfvén Mach number. We then extend and complete the analysis by covering other SW parameters such as velocity, magnetic field strength, density, dynamic pressure (defined as *p*_*dyn*_ = 0.5*ρv* ^2^), plasma beta, magnetosonic Mach number, and temperature and interpret the results.

## 2 Data

We compare in situ SW plasma and magnetic field data from OMNI during times when jets are observed with the SW measured during CMEs and as a reference during all times when magnetosheath data were available. We use 1‐min resolution OMNI velocity, magnetic field, and density data. Our data covers the time range between January 2008 and December 2021.

Data from the THEMIS spacecraft (Angelopoulos, 2008) are used to detect intervals of jets in the magnetosheath. Specifically, we use the reduced ion moments (ion velocity, density, temperature, and energy flux) from the THEMIS Electrostatic Analyzer (ESA; McFadden et al., 2008). Ion moments are available from ESA with a time resolution of 3 s. We use magnetic field measurements from the Fluxgate Magnetometer (FGM; Auster et al., 2008).

Magnetosheath intervals are determined by the same criteria used in Plaschke et al. (2013) and Koller et al. (2022): The spacecraft GSE position is restricted to 7–18 R_*e*_ and has to be within a 30° Sun‐centered cone with tip at the Earth. To ensure that the spacecraft is within the magnetosheath, the ion density has to be at least twice as dense as the upstream SW. The energy flux of the 10 keV ions has to be less than those of the 1 keV ions. The magnetosheath intervals are required to be longer than 2 min. All measurements are interpolated to the same 1 s time cadence for the subsequent detection of magnetosheath jets.

Jets were defined using the criteria of (Koller et al., 2022): $p_{\text{dyn,x}}>3\times {\langle p_{\text{dyn,x}}\rangle }_{20\text{min}}$. Here, ${\langle p_{\text{dyn,x}}\rangle }_{20\text{min}}$ denotes the 20‐min running average of the magnetosheath dynamic pressure in GSE‐X direction. Therefore, enhancements of the GSE‐X dynamic pressure larger than three times of the surrounding plasma within 20 min are declared as jets. As additional criterion, the jet velocity has to be negative in GSE‐X, and the magnetosheath GSE‐X velocity 1 min before and after the jet has to be above half of the measured GSE‐X velocity during the pressure peak of the jet. Magnetosheath intervals shorter than 20 min are not considered for jet detection. Jets were restricted to only those with a duration of more than 10 s. Using these criteria, we detected a total of 9704 jets within the given time range and the defined subsolar region of the magnetosheath. The number is smaller in comparison to the work by Koller et al. (2022) due to the jet duration restriction of 10 s and due to the usage of the reduced ion moments. The intervals of magnetosheath and jet times are provided at https://osf.io/s32yf/ (Koller et al., 2023).

Arrival times of ICMEs at Earth are collected from an online catalog maintained by Richardson and Cane (Cane & Richardson, 2003; Richardson & Cane, 2010). It includes a variety of information on near‐Earth CMEs that have been detected since 1996. We use the start and end times of CME‐MEs (labeled as ICME Plasma/Field Start, End) in our work, which are the times that were measured by ACE. We also use the time of the associated geomagnetic storm sudden commencement at Earth, which is associated with the arrival of the CME‐driven shock. We define the interval between the shock arrival and the start of the CME‐ME as the crossing time of the CME‐sheath, covering both compression regions, sheath and LE.

The list of SIR and HSS events described in Koller et al. (2022) was used in our work. It covers the start and end times of SIRs and HSSs from 1995 until the end of 2021 based on previously published lists (Geyer et al., 2021; Grandin et al., 2019; Jian et al., 2011) which were then extended. The start times of SIRs is defined as the start of increase in density and velocity, and its end is given by the time of the peak velocity. The HSS interval time is defined by the time between the peak SW velocity and the time, when the velocity drops below the defined threshold of each list as described in Koller et al. (2022).

## 3 Analysis

We use OMNI data during jet intervals and during all times when we have simultaneously magnetosheath observations by THEMIS. The latter is used as a reference to determine, how the SW parameters are distributed during jet detection times. For each second of magnetosheath data, we calculate the corresponding SW Alfvén Mach number and the cone angle. Both THEMIS ion moments and OMNI data are interpolated to 1 s resolution for that purpose. We start the analysis with these parameters as they directly influence the existence of the foreshock. To check how important these parameters are for the jet occurrence, we plot a 2‐dimensional (2D) histogram with the cone angle on the *x*‐axis and the Alfvén Mach number on the *y*‐axis. All histograms are normalized to the peak value. Bin sizes of 4.286° for the *x*‐axis and 1.3 for the *y*‐axis were chosen. These bin sizes ensure reliable amounts of data as well as reasonable resolution for our analysis.

We then determine the jet probability distribution as a function of Alfvén Mach number and cone angle. We do this by dividing the SW conditions that we find during jets by the overall SW conditions. The jet probability distributions shows, how the jet occurrence is related to the physical parameters, while the measured distribution during jets shows a bias toward parameter ranges with the most measurements. The result is a 2D histogram plot, where the jet probability given in percent is color‐coded in each bin. As a final analysis we check, how this jet probability distribution compares to the SW conditions that we find within CME‐sheaths, CME‐MEs, SIRs and HSSs.

The analysis is extended to a wider set of SW parameters by using 1D histograms similar to LaMoury et al. (2021) and Plaschke et al. (2013). We investigate how the conditions differ in each type of SW structure as well as in the non‐structured SW. We compare these conditions with the jet probability in percent for each SW parameter using histograms. We show the correlation matrix of the SW parameter to give context to the dependence of every parameter with each other.

We give uncertainty estimations for the jet probability in both 2D histograms and 1D histograms. We use bootstrapping to infer the uncertainties and to check, how robust the results are: A random subsample of the overall distribution is taken and the jet occurrence is evaluated with this subsample. This process is repeated 100 times, resulting in 100 different results. For each bin, we take the standard deviation of the resulting range as our measure of error. The size of the random subsample is 1/300th of the original dataset. Our dataset is using 1 min‐OMNI values that were interpolated to a resolution of 1 s. Therefore, a random subsample of 1/300th of the original sample size roughly corresponds to 20% of the measured OMNI distribution. The uncertainties are displayed as an additional plot for the 2D histograms and as errrorbars in the 1D histograms.

## 4 Results

**Figure 3:**
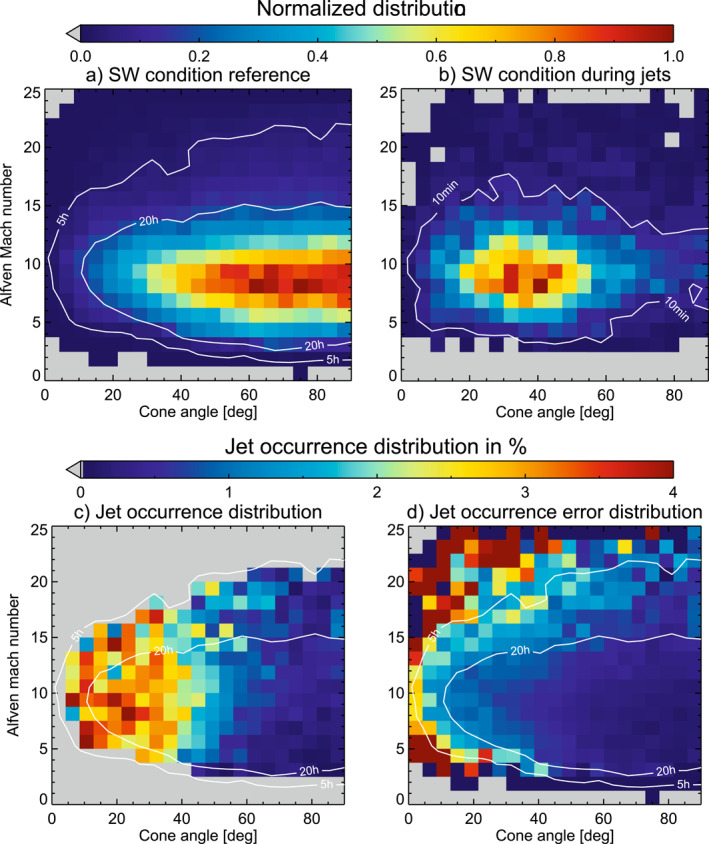
2D histogram showing normalized distributions of cone angle and Alfvén Mach number. Plot (a) shows the overall distribution of both parameter in the solar wind (SW) during all observation times. Plot (b) shows the SW parameter distribution during jet detection. Plot (c) shows the jet probability depending on both parameters, where the percentage of detecting a jet is color coded. The data was restricted to all times with more than 5 hr of available data, as indicated by the white contour in panel (a). Plot (d) shows the uncertainty estimation for each bin in plot (c) using the same range of value.

Figure 3a shows the 2D histogram distribution for the SW condition during all times when we have magnetosheath observations. The SW condition peak at cone angles of 40–90° and Alfvén Mach numbers around 5–12. This distribution serves as a reference for the further analysis. The white contour indicates all bins where we have more than 5 hr/20 hr of measurements available. The distribution of SW conditions during jets is shown in Figure 3b. We find that jets appear dominantly during cone angles of 20–50 and Alfvén Mach numbers of 6–11.

Figure 3c shows the jet probability in percent. Here, the distribution of SW conditions during jets is divided by the reference SW distribution. All bins with less than 5 hr of data outside of the white contour in Figure 3a were omitted. Table 7 in Koller et al. (2022) gives a mean value of roughly 1–3 jets per hour. Therefore, we can expect at least 5–15 jets for each bin within the given 5 hr contour. Figure 3d shows the corresponding uncertainties for each bin of Figure 3c. As expected, jets are found predominantly at lower cone angles, mostly at values lower than 40°. Jets are rarely detected during intervals with high cone angles (>60°). The jet probability decreases drastically for cone angles between 40–60°, with a slight trend of decreasing jet percentage for lower Alfvén Mach numbers. During these conditions, the probability to detect jets is roughly 9–15 times lower compared to times of low cone angle (<40°) and high Alfvén Mach numbers (>5). This value is similar or slightly higher to the probability of detecting jets downstream of the quasi‐parallel shock compared to the quasi‐perpendicular shock found by Archer and Horbury (2013). Figure 3c hints that the jet probability at low Alfvén Mach numbers (<5) is decreasing even for intermediate cone angles (40–50°), albeit with a low amount of data available in this regime.

**Figure 4:**
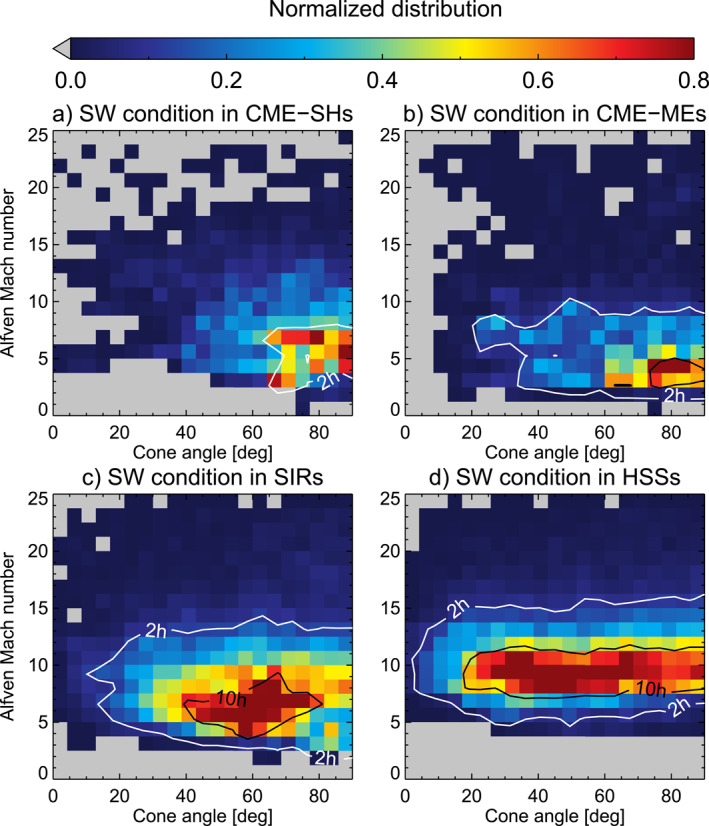
2D histogram showing normalized distributions of cone angle and Alfvén Mach number for each type of solar wind (SW) structure: (a) during coronal mass ejection (CME)‐sheaths, (b) during CME‐magnetic ejectas (MEs), (c) during stream interaction regions (SIRs) and (d) during high‐speed streams (HSSs). Contours indicate the number of available hours for each bin. The maximum value of the color scale is set to 0.8 to increase the visibility of the distribution.

This distribution is compared with the conditions found within SW structures given by Figure 4. Black contours in each panel indicate where we have more than 2 or 10 hr of available data per bin. Figure 4a shows the mean SW distribution for cone angle and Alfvén Mach number in CME‐sheaths. The distribution is mostly confined to cone angles higher than 65° and Alfvén Mach numbers between 3 and 9. It matches the area of lowest jet probability from Figure 3c.

The mean SW conditions that we can find during CME‐MEs is shown in Figure 4b. The same bin sizes from the previous plot were chosen. The distribution is mostly confined to cone angles higher than 60° and Alfvén Mach numbers between 2 and 5. It is similar to the CME‐driven sheaths, with the major difference being the lower Alfvén Mach numbers. It again matches with the parameter distribution least likely to show jet occurrence.

Figure 4c shows the mean SW distribution for cone angle and Alfvén Mach number for SIRs. The data shows a broader range compared to the distributions associated with CMEs. The distribution is confined mostly to the area at cone angles between 30 and 85° and Alfvén Mach numbers between 3 and 11.

Figure 4d shows the mean SW distribution for cone angle and Alfvén Mach number for HSSs. A narrow distribution is seen for the Alfvén Mach number, ranging from 6 to 13, while the cone angle covers a wide range with values between 15 and 90°. Comparing with the probability distribution for jets seen in Figure 3c, HSSs show the most overlap with conditions that are favorable for the jet generation.

**Figure 5:**
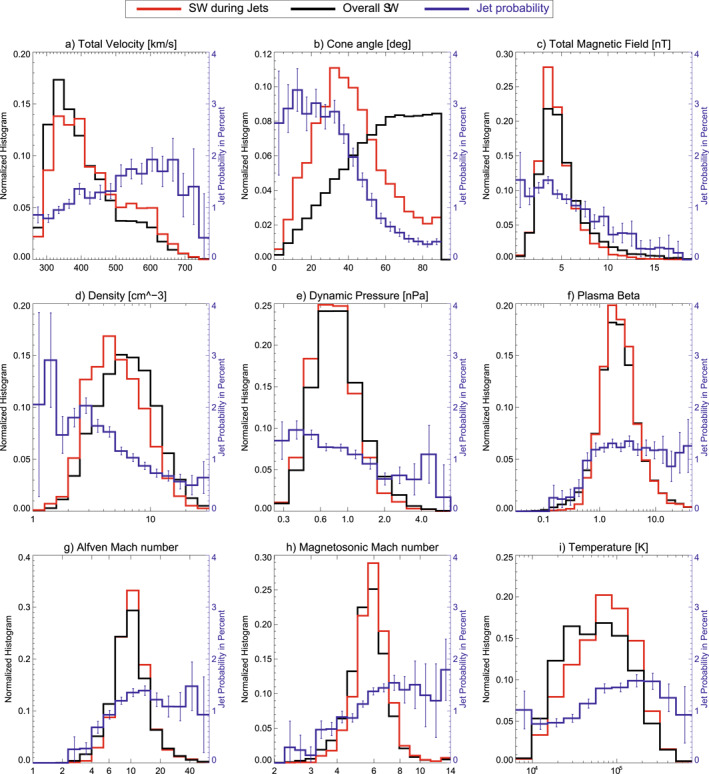
1D parameter histograms showing solar wind parameter distributions during jet detection (red) and during all available sheath times (black). The jet probability distribution in percent is given in blue. Estimations for the uncertainties in the jet probability distribution are derived using bootstrapping.

Figure 5 shows 1D histograms for other SW parameters when jets were detected, during all times of simultaneous magnetosheath measurements and the probability distribution for jet occurrence. The plasma beta in panel (f) is defined as *β* = 2*μ*_0_ *nk*_*B*_ *T*/*B* ^2^, with *μ*_0_ being the vacuum magnetic permeability, *n* the number density, *k*_*B*_ the Boltzmann constant, and *T* the temperature. The magnetosonic Mach number in panel (h) is defined as $M_{M}=v_{SW}/\sqrt{v_{A}^{2}+c_{s}^{2}}$, with *c*_*s*_ being the speed of sound in the SW. The *Y*‐Axis for the jet probability is the same for each panel in Figure 5. This helps to visualize and compare the impact of each parameter on the jet probability. Uncertainties for the jet probability are shown as errorbars in each panel. The trends for the jet probability at high and low values for each parameter are summarized in Table 1. The jet probabilities for a defined lower and upper range for each SW parameter are given. The cone angle shows the largest influence on the probability. Plasma beta, Alfvén Mach number, and magnetosonic Mach number see a drop in percentage at low values. A drop in jet percentage is notable for high magnetic field strengths. Both density and dynamic pressure have a higher jet percentage at lower values, while the total velocity shows a jet percentage increase at higher values. The SW temperature seems to be the parameter with the least influence, showing moderately higher jet percentages at high values. These results largely agree with the results given in LaMoury et al. (2021, dashed line in Figure 4).

**Table 1:** Mean Jet Probability at Low and High Ranges for Each Solar Wind Parameter Given in Figure 5

	Low range	Jet probability	High range	Jet probability
Total velocity	<400	1.0	>500	1.7
Cone angle	<40	2.8	>60	0.4
Total magnetic field	<6	1.3	>10	0.4
Density	<4	1.8	>10	0.6
Dynamic Pressure	<0.6	1.46	>2	0.7
Plasma beta	<0.5	0.3	>0.5	1.2
Alfvén Mach number	<5	0.5	>7	1.3
Magnetosonic Mach number	<3	0.2	>6	1.5
Temperature	<60,000	0.9	>60,000	1.5

**Figure 6:**
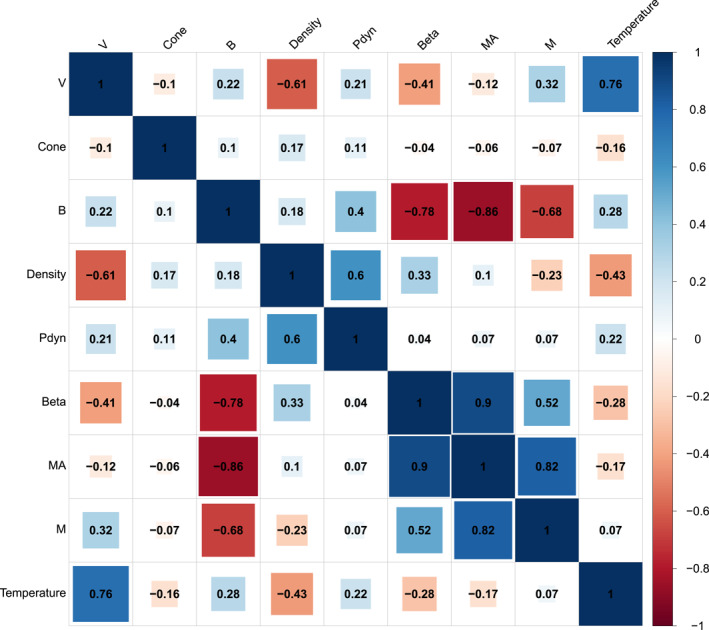
Spearman correlation coefficients for the nine solar wind parameters displayed in Figures 5, 6, 7, 8, 9. The correlation coefficients are calculated using OMNI data during all times of simultaneous magnetosheath measurements by THEMIS.

The Spearman correlation coefficients between each parameter in the SW is given in Figure 6 to give context to the previous figure. Some of these parameters are revealed to be highly correlated. This analysis uses the overall SW without differentiation between each structure and we derive some of well‐known relations. SW velocity is anti‐correlated with density (−0.61) and plasma beta (−0.41) and correlated with temperature (0.76), magnetosonic Mach number (0.32), magnetic field strength (0.22), and dynamic pressure (0.21). The cone angle has the weakest correlation with the other given parameters (0.17 with magnetic field strength, −0.16 for temperature, 0.11 for dynamic pressure and −0.1 for velocity). The magnetic field is anti‐correlated with the Alfvén Mach number (−0.86), plasma beta (−0.78), and magnetosonic Mach number (−0.68) and shows some correlation with dynamic pressure (0.4) and temperature (0.28). The SW density correlates with dynamic pressure (0.6) and plasma beta (0.33) and is anti‐correlated with temperature (−0.43) and magnetosonic Mach number (−0.23). Plasma beta is strongly correlated with the Alfvén Mach number (0.9), correlates with magnetosonic Mach number (0.52), and is anti‐correlated with temperature (−0.28). The Alfvén Mach number correlates with the magnetosonic Mach number (0.82).

**Figure 7:**
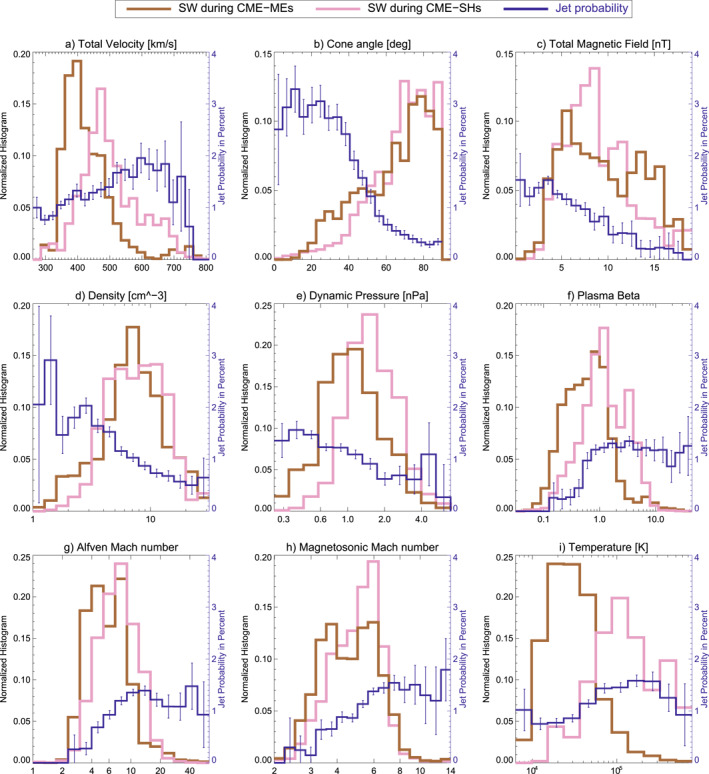
1D parameter histograms for the solar wind distribution in coronal mass ejection (CME)‐sheaths and CME‐magnetic ejecta (ME) compared to the overall jet probability distribution.

**Figure 8:**
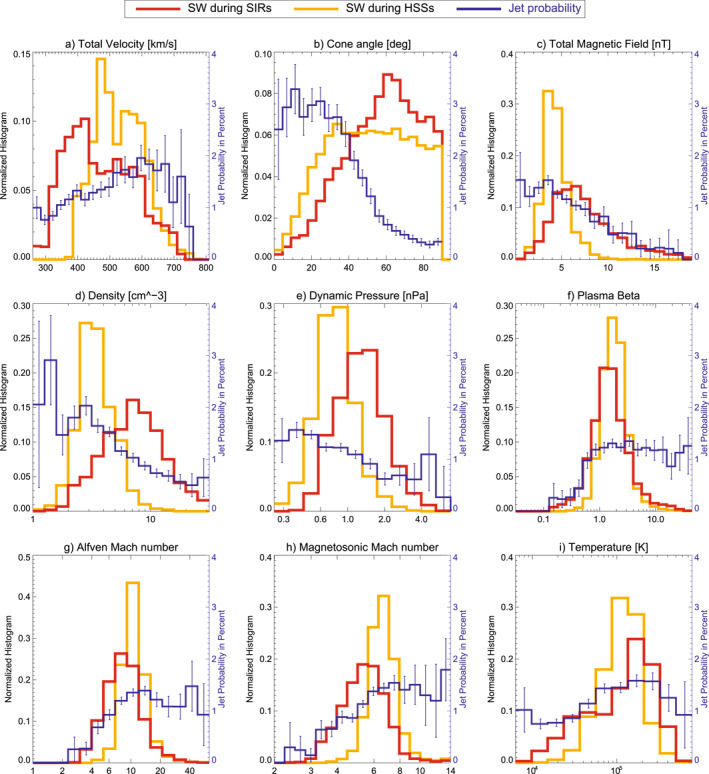
1D parameter histograms for the solar wind distribution in stream interaction regions and high‐speed streams compared to the overall jet probability distribution.

**Figure 9:**
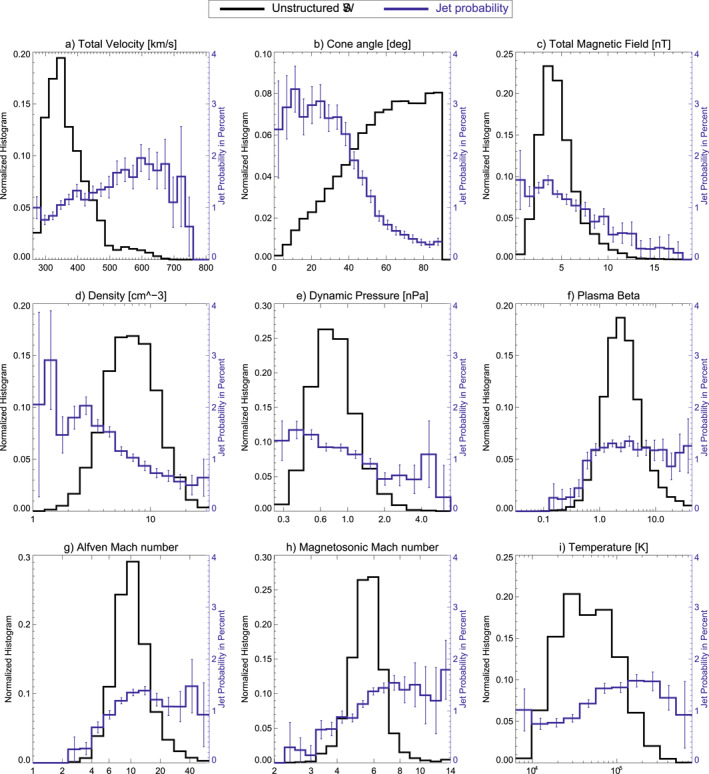
1D parameter histograms for the solar wind (SW) distribution during non‐structured SW compared to the overall jet probability distribution.

Figures 7 and 8 show 1D histograms for SW parameter during each type of large‐scale SW structure analyzed in this work. In addition to that, Figure 9 shows the distribution for non‐structured SW. The overall jet probability from Figure 5 is displayed in each case to connect the parameters with the jet occurrence.

Histograms of CME‐ME and CME‐sheath show a variety of parameter distributions unfavorable for jet occurrence as seen in Figure 7. Particularly, their high cone angles, high magnetic field strength, low plasma beta, and low Alfvén Mach number match with the decrease in jet occurrence. The velocity is increased in both structures (especially in the CME‐sheath) compared to mean SW conditions, which usually favors jet occurrence. CME‐sheaths show higher dynamic pressure than CME‐MEs, which is statistically less favorable for jets. On the other hand, CME‐MEs have less favorable conditions in plasma beta, Alfvén Mach number, and temperature compared to CME‐sheaths.

Figure 8 shows the distribution of parameters found in HSSs and SIRs. The parameter distribution in HSSs in particular co‐align with an increase in jet occurrence in every single parameter. In particular the tendency toward low cone angles, the consistent low magnetic field strength, low density, and high velocity results in a higher jet occurrence in HSSs compared to every other SW structure. SIRs show favorable conditions for jets in velocity and temperature compared to the mean SW. However, the cone angle distribution is skewed toward higher angles and the magnetic field, density, and dynamic pressure are increased, which is less favorable for jet occurrence.

The distribution of non‐structured SW is given in Figure 9. It mostly shows the slow SW because all time periods of high velocity come from structured SW. The unstructured SW shows more favorable distributions for jet generation in several parameters compared to some other SW structures. The distribution in cone angle, magnetic field strength, dynamic pressure, plasma beta, Alfvén Mach number, and magnetosonic Mach number favors jet occurrence during quiet SW times over CME‐MEs and CME‐sheaths. Slow SW, high density, high cone angle and low temperature show less favorable distributions for non‐structured SW.

## 5 Discussion and Conclusion

We analyze in detail how the distinct conditions within SW large‐scale structures such as SIRs and CMEs influence the parameters necessary to generate jets efficiently. According to the results in Figure 8, HSSs coming from solar coronal holes appear to be the ideal condition under which jets can form. They have a high probability to show low cone angles, high velocity, sufficiently high Alfvén Mach number and plasma beta, low magnetic field strength, and low density. In fact, numerous case studies analyzed magnetosheath jets that were generated during times of SW HSSs (see Archer et al., 2019; Escoubet et al., 2020; Hietala et al., 2009, 2012, 2018; Nykyri et al., 2019; Němeček et al., 1998; Raptis, Karlsson, Vaivads, Lindberg, et al., 2022). The SW parameter distributions in HSSs are very narrow by definition due to the high SW speeds and consequently low SW density. HSSs can have long durations and are rather stable, lasting for several days.

SIRs show a wide range of SW conditions due to our definition of their start‐and end time: they start at the onset of density + velocity increase and end at the peak of SW velocity. Within this time, the density increases to a sharp peak and drops down to a minimum value, which should roughly coincide with the peak velocity. Therefore, these time ranges cover a range of density from low to very high values and velocities from quiet SW up to the peak velocity. With the compressed SW, an increase in magnetic field strength is expected as well as an increase in temperature due to the compression. The cone angle distribution, especially below 45°, is similar to non‐structured SW, thus the overall effect on jet generation will be governed by other parameters. The increased velocity in SIRs favor jet generation, while the increased mean magnetic field strength works against it. The net effect on jet generation based on these statistics appears to be roughly zero, which is in agreement with the findings shown in Koller et al. (2022). There is still the possibility to generate jets through sudden changes in the orientation of the magnetic field due to the compression region, which needs further investigation using case studies.

The lowest number of jets is found within CME‐MEs (Koller et al., 2022) and can be explained by the high cone angle that renders the building of a foreshock difficult. With Θ_*Bn*_ (and as proxy the cone angle) having the most influence on the jet production, the foreshock would build up at positions far away from the Earth‐Sun line (as sketched in Figure 1 by Vuorinen et al., 2019). The Foreshock and its properties have been observed at positions far away from the subsolar region in recent studies (Turner et al., 2020; Vu et al., 2022) as well as simulations (e.g., in Palmroth et al., 2018a). In addition to this, sufficiently weak Alfvén Mach numbers might hinder the backstreaming of ions and thus the building of the foreshock and the reformation of the quasi‐parallel bow shock. Alfvén Mach numbers below 5 are rare considering the overall distribution of the SW. However, low Alfvén Mach numbers are very common conditions found within CME‐MEs. Turc et al. (2018) analyzed foreshock properties during enhanced IMF strength similar to CME‐MEs in hybrid‐Vlasov simulations. They found that foreshock structures have smaller scales in CME‐ME‐ like conditions. They also discuss that the shock rippling may occur at smaller scales as well, which directly connects to proposed jet generation mechanisms. Our findings regarding the jet occurrence in relation to SW conditions are further supported by simulation results done recently by Tinoco‐Arenas et al. (2022). The appearance of jets ceased at shocks with very low Alfvén Mach numbers. Similarly, high Θ_*Bn*_ angles (here as a proxy we use the cone angle at the subsolar point) caused a reduction of jet production in their simulations.

A higher number of jets in CME‐sheaths compared to CME‐MEs are reported in Koller et al. (2022). However, both CME‐sheath and CME‐ME show cone angles dominantly in the range of 70–90°. In fact, the cone angle distribution for CME‐sheaths is even more inclined toward high cone angles than CME‐MEs as seen in Figure 7. This indicates that other parameters dominate the enhanced jet generation within CME‐sheaths. Differences between CME‐sheath and CME‐ME are found for the mean Alfvén Mach number, plasma beta, velocity, and temperature that are higher for CME‐sheaths. The jets during CME‐sheaths might also be caused by the rapidly changing cone angle due to the turbulent compressed plasma, which might cause pressure pulses in the magnetosheath similar to the ones proposed in Archer et al. (2012). However, this needs further investigation in specific case studies.

As all events are restricted to times where THEMIS spacecraft were in the magnetosheath, the orbit and time of the year is an implicit factor in the given distribution of the SW parameters. CME‐MEs and CME‐sheath are more short‐lived compared to SIRs and HSSs, which reduces the amount of data for the analysis (see Table 1 in Koller et al., 2022). This might explain statistical differences between the parameter distribution given in this work compared to other statistical works on SW parameters (Yermolaev et al., 2021).

While the number of detected jets is significantly lower within CMEs (Koller et al., 2022), there is still a non‐vanishing amount of them. Whether CME generated jets are different compared to those occurring during low‐ cone angle and high‐Alfvén conditions will give more insight in their generation mechanisms. According to Hietala and Plaschke (2013), 97% of all jets are generated by the bow shock rippling at the quasi‐parallel shock. Kajdič et al. (2021) discussed jets that appeared behind the quasi‐perpendicular shock. They showed four different cases: magnetic flux tubes in the magnetosheath that are connected to the quasi‐parallel section of the shock, nonreconnecting current sheets at its upstream edge, reconnection exhausts due to a single IMF discontinuity, and mirror‐mode waves. The overall probability distribution of jets that were only detected during CME‐MEs (not shown) follows the same probability distribution as shown in Figure 3c. The only significant difference being that the favorable conditions for the jet generation are rarer within CME‐MEs. By using a large dataset of plasma measurements that spans over more than a decade, there is also the possibility that some of these residual jets are caused by uncertainties or errors in the data. The detection threshold is based on a 20 min average value and might introduce a small number of jets in case of sudden changes in the dynamic pressure within the magnetosheath. The OMNI data might have errors or might not match the values that were measured at the spacecraft position in the magnetosheath. This would result in inaccurate SW conditions during jet detection. In‐depth analysis of jet properties during CME‐MEs are needed to resolve individual causes for jets behind the quasi‐parallel shock.

At the planet Mercury, we also find low Alfvén Mach numbers similar to what we find within CME‐MEs at 1 AU. Karlsson et al. (2016) analyzed isolated magnetic field structures within the Hermean magnetosheath (Anderson et al., 2010) as possible analogs to terrestrial jets. However, the analyzed structures had no dependence on the Θ_*Bn*_ distribution, and without sufficient ion moment measurements the connection to the classical magnetosheath jets detected at Earth remains uncertain. Sundberg et al. (2015) suggested that at low Mach numbers the backstreaming ions might not be sufficient for the self‐reformation of the quasi‐parallel shock, and therefore prohibits the formation of strongly non‐linear structures like SLAMS. This could be similar to what we see at the Earth's bow shock during CME‐MEs. Based on our result, we postulate that the number of jets within the Hermean magnetosheath would be low. The BepiColombo mission will insert into an orbit around Mercury between December 2025 and March 2026 (Milillo et al., 2020). This mission will give new insights on the jet occurrence and generation at the Hermean magnetosheath and foreshock.

In summary, we show the distribution of SW parameters in CME‐MEs, CME‐sheaths, SIRs, and HSSs in the context of parameters that influence the jet generation. The mix of high cone angles and low Mach numbers are unfavorable SW conditions, hence, decreasing the production of jets in the magnetosheath. The condition within CME‐MEs is similar to this condition, which gives context to the low detection number of jets in this structure as was reported by Koller et al. (2022). Without a foreshock that generates strongly non‐linear structures near the subsolar region, the proposed jet generation mechanisms for the majority of jets is not applicable. Further investigation into the exact details is necessary to conclude, how the CME is disrupting the foreshock. Future case studies as well as simulations on the interaction of CMEs with the bow shock can complement our statistical work. The SW conditions that govern jet occurrence in SIRs, HSSs and unstructured SW are mostly governed by cone angle, density, and magnetic field. HSSs show the highest probability of jet occurrence in every SW parameter compared to all other SW structures, which is in agreement with the results by Koller et al. (2022). A next step is to analyze, whether the jets found during different structures have statistically distinctive differences in their properties. Cluster analysis using several SW parameters could give more insights in which combination of SW parameters can generate jets.

## Data Availability

We thank C. W. Carlson and J. P. McFadden for use of ESA data. We acknowledge the use of NASA/GSFC's Space Physics Data Facility's OMNI data and web services (https://omniweb.gsfc.nasa.gov/html/omni_min_data.html). THEMIS and OMNI data were accessed using the SPEDAS software (Angelopoulos et al., 2019). We provide the jet lists as well as the magnetosheath times at https://osf.io/s32yf/.
